# *Pseudoalteromonas piratica* strain OCN003 is a coral pathogen that causes a switch from chronic to acute *Montipora* white syndrome in *Montipora capitata*

**DOI:** 10.1371/journal.pone.0188319

**Published:** 2017-11-16

**Authors:** Silvia Beurmann, Blake Ushijima, Patrick Videau, Christina Marie Svoboda, Ashley Marie Smith, Orion Silverstar Rivers, Greta Smith Aeby, Sean Michael Callahan

**Affiliations:** 1 Universtiy of Hawaiʻi at Mānoa, Department of Microbiology, Honolulu, HI, United States of America; 2 Hawaiʻi Institute of Marine Biology, Kāneʻohe, HI, United States of America; 3 Oregon State University, College of Veterinary Medicine, Corvallis, OR, United States of America; 4 Dakota State University, College of Arts and Sciences, Madison, SD, United States of America; The Rockefeller University, UNITED STATES

## Abstract

Reports of mass coral mortality from disease have increased over the last two decades. *Montipora* white syndrome (MWS) is a tissue loss disease that has negatively impacted populations of the coral *Montipora capitata* in Kāne‘ohe Bay, Hawai‘i. Two types of MWS have been documented; a progressive disease termed chronic MWS (cMWS), that can be caused by *Vibrio owensii* strain OCN002, and a comparatively faster disease termed acute MWS (aMWS), that can be caused by *Vibrio coralliilyticus* strain OCN008. *M*. *capitata* colonies exhibiting cMWS can spontaneously switch to aMWS in the field. In this study, a novel *Pseudoalteromonas* species, *P*. *piratica* strain OCN003, fulfilled Koch’s postulates of disease causation as another etiological agent of aMWS. Additionally, OCN003 induced a switch from cMWS to aMWS on *M*. *capitata* in laboratory infection trials. A comparison of OCN003 and *Vibrio coralliilyticus* strain OCN008, showed that OCN003 was more effective at inducing the cMWS to aMWS switch in *M*. *capitata* than OCN008. This study is the first to demonstrate that similar disease signs on one coral species (aMWS on *M*. *capitata*) can be caused by multiple pathogens, and describes the first *Pseudoalteromonas* species that infects coral.

## Introduction

Coral disease is an environmental and economic concern that threatens the continued survival of coral reef ecosystems and intensifies the impacts of other recognized detriments to coral health. Following the first report of coral disease in 1973 [1], over 40 coral diseases from at least 75 countries have been described and characterized [2]. More than 200 species of reef-building corals are affected by different diseases, and reports of mass mortality events have been increasing over the last two decades [3–7]. For many of these diseases, a causative agent remains to be found.

Koch’s postulates of disease causation are a set of guidelines that are commonly used to infer the causality of a microorganism as a disease agent [8]: (I) The microorganism must be present in all organisms that have the disease and should be absent in healthy organisms, (II) the microorganism should be isolated from a diseased organism and can be grown in pure culture, (III) the cultured microorganism should cause disease signs if inoculated into healthy hosts, and (IV) the same microorganism must be re-isolated from the inoculated, diseased hosts. These postulates have been utilized by researchers to describe causative agents of coral diseases, although only seven causal relationships linking pathogens to a coral disease have been successfully demonstrated to date [9–15].

The fulfillment of Koch’s postulates has proven useful in organismal studies, but the advent of genetic analysis has prompted the repurposing of these postulates for use in molecular pathogenesis research. Using an analogous line of reasoning to organismal investigations, studies must fulfill Koch's molecular postulates to assess the role of a gene in a pathogen's virulence [16]. Like Koch’s postulates, for a gene to be considered a virulence factor, mutations abrogating gene function lead to decreased virulence of the pathogen. Complementation of the mutation via reintroduction of the gene to restore function results in a concurrent return to wild type virulence levels. To date, only a handful of genes have been implicated as virulence determinants in coral pathogens. A toxin and a superoxide dismutase have been shown to be virulence factors produced by the pathogen *Vibrio shiloi*, which causes coral bleaching of *Oculina patagonica* [17,18]. Another study demonstrated that a metalloprotease produced by *V*. *coralliilyticus* strains BAA-450 and P1-4 caused tissue lesions in *Pocillopora damicornis* and *Acropora millepora* coral colonies, respectively [19,20]. In a recent study, mutation of a toxin regulator (*toxR*) and a gene within the type IV pilus operon (*mshA*) in *V*. *coralliilyticus* strains OCN008 and OCN014 significantly reduced infection rates in the coral *M*. *capitata* and *Acropora cytherea*, respectively [15]. While such studies on the molecular basis of pathogenesis have been conducted extensively in human pathogens, similar work is almost entirely lacking from coral disease research.

*Montipora* white syndrome (MWS) is a tissue loss disease that has negatively impacted populations of *Montipora capitata*, a major reef-building coral in Kāne‘ohe Bay, Hawai‘i [21]. Two types of MWS have been documented; a chronic, progressive disease that displays diffuse tissue loss termed chronic MWS (cMWS) [13], and a comparatively faster progressive tissue loss disease termed acute MWS (aMWS) [7,14]. *Montipora capitata* colonies exhibiting cMWS are observed at consistent levels throughout the year [21], while outbreaks of aMWS occur sporadically usually during the rainy, winter months [7]. Colonies with cMWS can survive months to years because of the comparably slower rate of tissue loss [21]. In contrast, aMWS-affected colonies can die within days to weeks during disease outbreaks [7]. During aMWS outbreaks, disease transmission has been observed between neighboring *M*. *capitata* colonies in the field, and cMWS-affected colonies were also observed to spontaneously switch to aMWS, which can lead to complete colony mortality [7]. While this switch from cMWS to aMWS has been observed both in laboratory infection trials and on coral colonies in the field [13,22,23], the mechanism underlying the change in disease signs remains unknown.

To date, two pathogenic *Vibrio* species have been shown to induce disease signs consistent with MWS in *M*. *capitata*; *Vibrio owensii* strain OCN002 induces cMWS and *Vibrio coralliilyticus* strain OCN008 induces aMWS (hereafter referred to as OCN002 and OCN008) [13,14]. During controlled laboratory infections of *M*. *capitata*, OCN002 caused cMWS in 53% of inoculated coral fragments in an average of 28 days post-inoculation [13], while OCN008 infected 80 to 100% of fragments resulting in aMWS within four days post-inoculation [14,15]. A comparison of the culturable bacterial communities from healthy and aMWS-affected *M*. *capitata* from Kāne‘ohe Bay found that *Pseudoalteromonas* was the second most abundant bacterial genus in infected tissue (second to *Vibrio*) but was absent from healthy mucus [24]. Based on these findings, a *Pseudoalteromonas* strain isolated from aMWS-affected *M*. *capitata* was investigated as another potential pathogen. The *Pseudoalteromonas* strain originally described by Smith (2008) has recently been identified as a novel *Pseudoalteromonas* species, *P*. *piratica* strain OCN003 [25], which was assessed as another etiological agent of acute tissue loss disease in *M*. *capitata* (aMWS).

In this study, Koch's postulates of disease causation were fulfilled for *P*. *piratica* strain OCN003, which establishes it as another etiological agent of aMWS in *M*. *capitata*. In addition, when inoculated onto coral fragments exhibiting cMWS signs, OCN003 also induced the switch from cMWS to aMWS in *M*. *capitata* and again fulfilled Koch’s postulates as an etiological agent for this type of infection. To show that the switch from cMWS to aMWS was due to inoculation with wild type OCN003, and demonstrate that motility is a critical component of the infection process, a non-motile Δ*fliF*::*bla* mutant of OCN003 was constructed and found to be incapable of inducing the switch from cMWS to aMWS. Complementation of the non-motile OCN003 mutant restored the bacterium's ability to cause the switch from cMWS to aMWS, fulfilling Koch's molecular postulates of disease causation [16]. This work describes both the first strain of *Pseudoalteromonas* that infects coral and the first coral pathogen that can induce a switch in disease signs from a chronic (cMWS) to an acute (aMWS) type of tissue loss disease.

## Materials and methods

### Bacterial growth conditions

All bacterial strains used in this study are listed in Table 1. All marine bacterial strains were grown at 27°C in glycerol artificial seawater (GASW) broth or on plates solidified with 1.5% (w/v) agar as previously described [13]. *Escherichia coli* strains were grown in LB-Miller medium and incubated at 37°C. Antibiotics for plasmid selection in *E*. *coli* were used at the following concentrations: ampicillin, 100 μg/ml; kanamycin, 50 μg/ml; streptomycin, 50 μg/ml; spectinomycin, 100 μg/ml; and chloramphenicol, 30 μg/ml. Auxotrophic *E*. *coli* strains π3813 and β3914 were grown on media supplemented with deoxythymidine (DT) or diaminopimelate (DAP) at a final concentration of 0.3 mM each, respectively [26]. Bacterial *sacB*-mediated counterselection was achieved by supplementing GASW with 5% (w/v) sucrose [27].

**Table 1:** Strains and plasmids used in this study.

Strain or plasmid	Relevant characteristic(s)	Source or citation
***Pseudoalteromonas piratica* strains**		
OCN003	Wild type; isolated from the mucus of diseased *M*.*capitata*; Km^r^	Beurmann *et al*., 2015
OCN050	Wild type; isolated from diseased *M*.*capitata* during aMWS outbreak; Km^r^	This study
OCN051	Wild type; isolated from diseased *M*.*capitata* during aMWS outbreak; Km^r^	This study
OCN052	Wild type; isolated from diseased *M*.*capitata* during aMWS outbreak; Km^r^	This study
OCN003 Δ*fliF*::*bla*	OCN003 Δ*fliF*::*bla* mutant; Ap^r^, Km^r^	This study
**Marine bacterial strains**		
OCN004	Non-pathogenic *Alteromonas* sp; negative-control bacterium	Ushijima *et al*., 2012
OCN008	Pathogenic *Vibrio coralliilyticus* strain; Ap^r^	Ushijima *et al*., 2014
***Escherichia coli* strains**		
β3914	Δ*dapA*::(*erm-pir*); Km^r^, Em^r^, Tc^r^	Le Roux *et al*., 2007
π3813	Δ*thyA*::(*erm-pir*); Em^r^	Le Roux *et al*., 2007
DH5α	F- supE44 ΔlacU169 (φ80lacZΔM15) ΔargF hsdR17 recA1 endA1 gyrA96 thi-1 relA1	Stratagene
**Plasmids**		
pBBR1MCS-4	Source of Ap^r^ cassette; Ap^r^	Kovach *et al*., 1994
pBluescript SK+	Cloning vector, Ap^r^	Stratagene
pEVS78	Shuttle vector; Cm^r^	Stabb and Ruby, 2002
pRK2013	Self-transmissible vector for conjugation; Km^r^	Figurski and Helinski, 1979
pRL1383a	Replicative vector derived from RSF1010; Sp^r^, Sm^r^	Wolk *et al*., 2007
pRL277	Source of *sacB*; Sp^r^, Sm^r^	Black *et al*., 1993
pSW4426T	Source of Cm^r^, Sp^r^, and Sm^r^ cassettes; Cm^r^, Sp^r^, Sm^r^	Le Roux *et al*., 2007
pUC18-mini Tn7T-*lacZ*	Source of Gm^r^ cassette; Ap^r^, Gm^r^	Choi *et al*., 2005
pBU187	Modified version of pSW4426T without *ccdB* or *araC*; Cm^r^, Sp^r^, Sm^r^	This study
pSB122	Modified version of pBluescript SK+; Ap^r^, Cm^r^, Sp^r^, Sm^r^	This study
pSB123	Modified version of pBluescript SK+ with *sacB*; Ap^r^, Cm^r^, Sp^r^, Sm^r^	This study
pSB124	Modified version of pEVS78 with *fliF-*deletion construct; Cm^r^	This study
pSB125	Modified version of pEVS78 with *fliF-*deletion construct and Ap^r^ cassette; Cm^r^	This study
pSB126	Suicide vector used to create OCN003 Δ*fliF*::*bla* mutant; Ap^r^, Km^r^	This study
pSB127	pBluescript SK+ with *fliF*; Ap^r^	This study
pSB128	pBluescript SK+ with Gm^r^ and *fliF*; Ap^r^, Gm^r^	This study
pSB129	Replicative vector derived from pRL1383a; Sp^r^, Sm^r^, Gm^r^	This study

Abbreviation for antibiotic resistance cassettes: Ap^r^, ampicillin resistance; Sm^r^, streptomycin resistance; Sp^r^, spectinomycin resistance; Gm^r^, gentamicin resistance; Km^r^, kanamycin resistance; Em^r^, erythromycin resistance; Tc^r^, tetracycline resistance; Cm^r^, chloramphenicol resistance.

### Coral collection and infection trials

Healthy and cMWS-infected fragments of *M*. *capitata* were collected from the fringing reef surrounding Moku o Lo‘e island in south Kāne‘ohe Bay, Hawai‘i, under Special Activities Permits SAP#2013–47, SAP#2015–17, and SAP#2015–48 granted by the State of Hawai‘i, Department of Land and Natural Resources, Division of Aquatic Resources. Fragments with cMWS were identified by their characteristic lesions. Histology on the coral fragments was not performed, as the mucus associated bacteria are lost during sample processing, thus the specific causative agent of disease was not determined prior to experimentation. *M*. *capitata* fragments were allowed to recover from collection in a flow-through water table at ambient temperature for at least two days prior to experimental infection trials. Infection trials employed a block design in which all coral fragments used within an experimental block were collected from the same coral colony to control for intraspecific variability in disease susceptibility. Each experimental block consisted of one seawater control kept in a water table, one FSW control kept in its own tank, one bacterial control kept in its own tank, and any pathogen candidates tested.

Infection trials were conducted as previously described [13–15] with minor modifications (S1 Fig). Briefly, each *M*. *capitata* fragment with a cMWS lesion was individually housed in a nine-liter tank filled with seven liters of filtered seawater (FSW). Healthy *M*. *capitata* fragments were individually housed in four-liter tanks filled with three liters of FSW. All tanks were maintained at a constant 25°C water temperature. For coral inoculation, overnight liquid cultures of the various bacteria tested were diluted 1:1000 in GASW broth, and then grown to an OD_600_ (optical density measured at 600 nm) of 0.8 before being washed once and resuspended in autoclaved FSW. OCN003, the Δ*fliF*::*bla* mutant, the complemented Δ*fliF*::*bla* mutant, OCN004, OCN008, and OCN050 were inoculated to a final concentration of 10^8^ CFU/ml of tank water unless otherwise stated during infectious dose determinations. Dilutions were prepared as previously described in Ushijima *et al*. (2014). OCN004 was used as a negative control organism to show that the presence of a high concentration of bacteria in tank water was not sufficient to induce disease on *M*. *capitata* as previously described [13,14].

### Identification and phylogenetic analysis of *Pseudoalteromonas* isolates

*Pseudoalteromonas piratica* strain OCN003 was originally isolated as described in Smith (2008). To determine whether OCN003, or similar *P*. *piratica* strains, may have been involved in the March 2010 aMWS outbreak samples, bacteria were cultured from the mucus of infected colonies. Coral mucus was homogenized in FSW and serial dilutions were plated on GASW plates as previously described [13]. A total of 265 bacterial isolates were cultured from four aMWS-infected coral fragments collected during the outbreak. These isolates were screened by PCR using primers that selectively amplify a unique region of the OCN003 genome (003unique-F and 003unique-R). The unique region used for OCN003 identification was found by searching the genome [28] for a large intragenic region between convergent genes that was not similar to any sequences present in the NCBI database. All oligonucleotides used in this study are listed in Table 2. Nucleotide sequences for the 16S rRNA gene and the genes for the multilocus sequence analysis (MLSA) for 14 *Pseudoalteromonas* species were obtained from whole genome sequences from NCBI (Table 3). For MLSA, the *ftsZ*, *gapA*, and *recA* genes from *P*. *piratica* strains OCN003, OCN050, OCN051, and OCN052 were amplified by PCR using their respective primers, then sequenced with the same primers (Table 2), and then aligned and analyzed as previously described [25].

**Table 2:** Oligonucleotide primers used in this study.

Primer name	Primer sequence (5’ → 3’ orientation)	Source/citation
8F	AGAGTTTGA TCCTGGCTCAG	Aebischer *et al*., 2006
1513R	GGTTACCTTTGTTACGACTT	Aebischer *et al*., 2006
ftsZ-F	GTDATGTCWGCDATGGGHACNGCNATGATGGG	Sawabe *et al*., 2007
ftsZ-R	TGHTTRCGTAAAAAHGCNGGDATRTCHAARTARTC	Sawabe *et al*., 2007
gapA-F	GTNYTNTAYGGYTTTGGTCGYATYGGYCG	Sawabe *et al*., 2007
gapA-R	ACYTGRCAGCTRTARCCAAAYTCRTTRTCRTACCASAC	Sawabe *et al*., 2007
recA-F	CAAATTGARCGNCARTTTGGTAAAGGYTCAATYATG	Sawabe *et al*., 2007
recA-R	RTARCTRSACCASGCRCCNGCTTTSTCAAC	Sawabe *et al*., 2007
003unique-F	GCACACCTAGCTCATCTTCAAGCATACGTACTTG	This study
003unique-R	GGCCATCACCTAAGTTGTAATCCACTAAC	This study
pSW4426T-up-AraC-SacI	ATATATGAGCTCTTGGTAACGAATCAGACAATTGACGGCTTG	Ushijima *et al*., 2016
pSW4426T-down-ccdB	TCTGGGGAATATAAGAGCTCCAGCTTTTGT	Ushijima *et al*., 2016
M13-F	GTAAAACGACGGCCAGTG	Messing *et al*., 1983
M13-R	GGAAACAGCTATGACCATG	Messing *et al*., 1983
003-fliF-up-F	ATATATACTAGTATCGAGCAAGGTTACAGCGAGTGTTGTA	This study
003-fliF-OEX-R	TTGGTTCTCTTGATCAGTCATCTTCCCCGGGGGTGTCCATTGTTGTTAA	This study
003-fliF-down-OEX-F	ACAGATTTAACAACAATGGACACCCCCGGGGAAGATGACTGATCAAGAG	This study
003-fliF-down-R	ATATATACTAGTTCGCTTGAATACCGCGATCATCTACATC	This study
bla-F	ATATATCCCGGGAGCTGTTTCCTGTGTGA	This study
bla-R	ATATATCCCGGGAGCGCCAGCAGGAACGCGGGCGCGCA	This study
pEVS78-MCS-F	GCCCACCTATCAAGGTGTACTGCCTTCCAG	This study
pEVS78-MCS-R	CAAATGTAGCACCTGAAGTCAG	This study
003-fliF-F	TCTAGATAAGCGATAGTGGAGTAGGGTTGT	This study
003-fliF-R	GAGCTCGTCATCTTCGGTTAACCACGCTTTA	This study
Gm^R^-F	CTCGAGAAGATCCCCTGATTCCCTTTGTCAA	This study
Gm^R^-R	ATCGATTTAGGTGGCGGTACTTGGGTCGATA	This study
003-fliF-outside-F	CCATTCATACTGATGACATTTTCTTAGGC	This study
003-fliF-outside-R	CATTAATACGTCTTGCTGAACTTCACG	This study
pRL1383a-MCS-F	CGAAGTTATATTCGATGCGG	Ushijima *et al*., 2012
pRL1383a-MCS-R	CATTATGGTGAAAGTTGGAACC	Ushijima *et al*., 2012

**Table 3:** GenBank accession numbers for gene sequences and proteins used in this study.

Strain	16S rRNA gene	*recA*		*gapA*	*ftsZ*	
*Alteromonas macleodii* ATCC 27126^T^	CP003841	AFS36391		AFS37755	AFS38337	
*Pseudoalteromonas arctica* A 37-1-2^T^	DQ787199	ERG11012		ERG10585	ERG09017	
*Pseudoalteromonas atlantica* T6c^T^	CP000388	WP_011575995		WP_011575374	WP_011576244	
*Pseudoalteromonas citrea* NCIMB 1889^T^	X82137	ERG18422		ERG18058	ERG17458	
*Pseudoalteromonas flavipulchra* JG1	GU325751	WP_010607203		WP_010604051	WP_010605797	
*Pseudoalteromonas haloplanktis* ATCC 14393^T^	X67024	WP_016708515		WP_016707348	WP_016709471	
*Pseudoalteromonas luteoviolacea* ATCC 29581^T^	X82144	CCQ09551		CCQ12266	CCQ10341	
*Pseudoalteromonas marina* mano4^T^	AY563031	ERG27935		ERG27540	ERG27728	
*Pseudoalteromonas phenolica* KCTC 12086	AB607331	WP_058031021		WP_058029496	WP_058031515	
*Pseudoalteromonas piratica* OCN003^T^	KF042038	WP_038640125		WP_038641289	WP_038639463	
*Pseudoalteromonas piscicida* JCM 20779^T^	AB681918	ERG34167		ERG34147	ERG34433	
*Pseudoalteromonas rubra* ATCC 29570^T^	X82147	ERG44323		ERG44376	ERG46282	
*Pseudoalteromonas ruthenica* S3137	AF316891	KJY97116		KJY99687	KJY98742	
*Pseudoalteromonas spongiae* UST010723-006^T^	AY769918	ERG55196		ERG52548	ERG54604	
*Pseudoalteromonas tunicata* D2^T^	Z25522	EAR29741		EAR27888	EAR28847	

### Re-isolation of OCN003 and OCN050 from infected coral fragments

The re-isolation of OCN003 and OCN050 tagged with the non-self-transmissible vector pRL1383a [29] was conducted as previously described [13–15] with some modifications. Infected coral fragments were crushed and plated in dilutions on GASW plates supplemented with spectinomycin and streptomycin. Isolates that grew were screened using pRL1383a-MCS primers and the *P*. *piratica-*specific primers, 003unique-F and 003unique-R (Table 2). The *P*. *piratica-*specific primers were designed to amplify a 511 bp intergenic region in the OCN003 genome between the divergently expressed coding sequences of a putative response regulator [AIY65323], with 83% identity to chemotaxis protein CheY from *Pseudoalteromonas* sp. P1-9, and a Cys regulon transcriptional activator [AIY65324], with 99% identity to the transcriptional regulator CysB from *Pseudoalteromonas* sp. P1-9. According to BLAST analysis, the intergenic region did not share significant nucleotide similarity with any other sequence in the NCBI database. Positive colonies displayed amplified bands following amplification with both the pRL1383a- and *P*. *piratica-*specific primer pairs. In addition, the 16S rRNA gene was amplified and sequenced to ensure that recovered *P*. *piratica* isolates were all identical to the OCN003 and OCN050 stock cultures.

### Plasmid construction

All plasmids used in this study are listed in Table 1. Plasmid pBU187 is a suicide plasmid based on pSW4426T [26]. A fragment containing *araC*-PBAD-*ccdB* was removed from pSW4426T by amplifying it with PCR using the primers pSW4426T-up-AraC-SacI and pSW4426T-down-ccdB, digesting the product with *Sac*I and *Dpn*I, and then self-ligating it to create pBU187.

Plasmid pSB123 is a suicide plasmid based on pBU187 used to create genetic mutations in OCN003. The R6K *oriV*, *oriT*, and the chloramphenicol acetyltransferase gene (*cat*) were excised from pBU187 as a ~1.7 kb *Sac*I/*Xba*I fragment and cloned into the same sites of pBluescript SK+ (Stratagene) to create pSB122. The R6K *oriV*, *oriT*, *cat*, and multiple cloning site were amplified from pSB122 with PCR using the primers M13-F and M13-R, and the product was cloned into the *Eco*RV sites of pRL277 [30] to replace the existing *oriV* and *oriT* and create pSB123.

Plasmid pSB126 is a suicide vector based on pSB123 used to delete all but the first and last 18 nucleotides of the *fliF* coding region in OCN003. Regions up- and downstream of the *fli*F gene were amplified by PCR from OCN003 chromosomal DNA using the primers pairs 003-fliF-up-F and 003-fliF-OEX-R and 003-fliF-down-OEX-F and 003-fliF-down-R, respectively. The up- and downstream fragments were fused together by overlap extension PCR [31] and the product was cloned into the *Eco*RV site of the plasmid pEVS78 [32] to create pSB124. A fragment containing P_*bla*_-*bla* was amplified by PCR from pBBR1MCS-4 [33] with the primers bla-F and bla-R and cloned into the *Sma*I site in pSB124 to create pSB125. A fragment harboring regions up- and downstream of the *fli*F gene and P_*bla*_-*bla* was amplified from pSB125 by PCR using the primers pEVS78-MCS-F and pEVS78-MCS-R. The resulting product was digested with *Spe*I and cloned into the same site in pSB123 to create the vector pSB126.

Plasmid pSB129 is a replicative plasmid based on pRL1383a used to complement the OCN003 Δ*fliF*::*bla* mutant. The *fliF* gene was amplified by PCR from OCN003 chromosomal DNA using the primers 003-fliF-F and 003-fliF-R and ligated into the *Sma*I site of pBluescriptSK+ to create pSB127. The gentamicin resistance cassette was amplified by PCR from pUC18-mini-Tn7T-Gm-*lacZ* [34] using the primers Gm^R^-F and Gm^R^-R and cloned as a *Xho*I-*Cla*I fragment into the same sites of pSB127 to create pSB128. The gentamicin resistance cassette and *fliF* gene were excised from pSB128 as a *Sac*I and *Xho*I fragment and cloned into the same sites in pRL1383a to create pSB129.

### Strain creation and bacterial conjugation

The *fliF* gene was deleted from the OCN003 genome by allelic exchange with the suicide vector pSB126. The suicide vector pSB126 was introduced into OCN003 using tri-parental conjugation with *E*. *coli*. The suicide vector was maintained in *E*. *coli* strain β3914 [26] and the self-transmissible vector pRK2013 [35] was maintained in strain π3813 [26]. For tri-parental conjugation, the donor and recipient strains were grown overnight under antibiotic selection for plasmid maintenance and with DAP or DT as required. Overnight cultures were diluted 1:1000 in fresh culture medium, grown to an OD_600_ of 0.7, and washed three times with either GASW or LB-Miller for *Pseudoalteromonas* or *E*. *coli* strains, respectively. The strains were then resuspended in GASW to a total volume of 30 μl, combined, and spotted onto GASW plates supplemented with DAP and DT. Conjugation spots were incubated at 28°C for 24 h before being resuspended in GASW, washed three times with fresh GASW, and then dilutions were plated onto GASW agar supplemented with chloramphenicol, but lacking DAP or DT for counterselection against the auxotrophic *E*. *coli* donor strains. Chloramphenicol resistant colonies, which consisted of bacteria with the suicide vector introduced through a single recombination event, were used to inoculate GASW broth and incubated for 15 h. After incubation, cultures were washed with GASW three times, and then dilutions were plated onto GASW agar supplemented with sucrose and ampicillin to isolate double-recombinant mutants. The resulting strain, OCN003 Δ*fliF*::*bla*, was verified by PCR with the primer pair 003-fliF-outside-F and 003-fliF-outside-R, which anneal outside the region of DNA used to make the mutations and sensitivity to chloramphenicol.

The complementation plasmid was introduced into the OCN003 Δ*fliF*::*bla* mutant via tri-parental conjugation as described above. Colonies that grew on GASW plates supplemented with gentamicin were confirmed by PCR using the primers pRL1383a-MCS-F and pRL1383a-MCS-R and then screened for motility using light microscopy.

### Microscopy

Motility of the OCN003 Δ*fliF*::*bla* mutant, the complemented OCN003 Δ*fliF*::*bla* mutant, and wild type OCN003 were determined using both light microscopy and a semi-solid assay (GASW broth and 0.15% agar) in poured plates for the differentiation of motile and non-motile colonies. Cell morphology of wild type OCN003, OCN003 Δ*fliF*::*bla*, and the complementation of OCN003 Δ*fliF*::*bla* were examined using transmission electron microscopy (TEM). Overnight cultures were deposited on a Formvar-coated copper grid, contrasted with 1% uranyl acetate, and viewed on a Hitachi HT7700 TEM at 100kV. The presence of a flagellum was examined and photographed with an AMT XR-41B 2k x 2k CCD camera.

### Biofilm assays

Biofilm formation of the wild type OCN003, OCN003 Δ*fliF*::*bla* mutant, and complemented OCN003 Δ*fliF*::*bla* mutant was tested using a microtiter dish biofilm formation assay as previously described [36] with minor modifications. Bacterial cells were grown overnight in GASW broth, 10 μl of an overnight culture was used to inoculate each microtiter dish well (24-well dish) containing two ml of sterile GASW broth. The microtiter dish was incubated for 24 to 96 h at 28°C. After incubation, the culture was aspirated and the wells were rinsed twice with deionized water. To stain the biofilm, two ml of a 0.1% crystal violet solution was added to each well and then incubated at room temperature for 15 min. The crystal violet solution was aspirated and then the plate was rinsed with water and dried overnight at room temperature. For the quantification of the biofilm, two ml of 30% acetic acid was added to each well to solubilize the crystal violet, and then the solution was transferred to a new microtiter plate and the absorbance was measured at 550 nm. The data was analyzed by one-way ANOVA, followed by Tukey's multiple comparison with no significantly different results.

## Results

### *Pseudoalteromonas piratica* strain OCN003 is another etiological agent of acute tissue loss lesions (aMWS) in *Montipora capitata*

**Fig 1:**
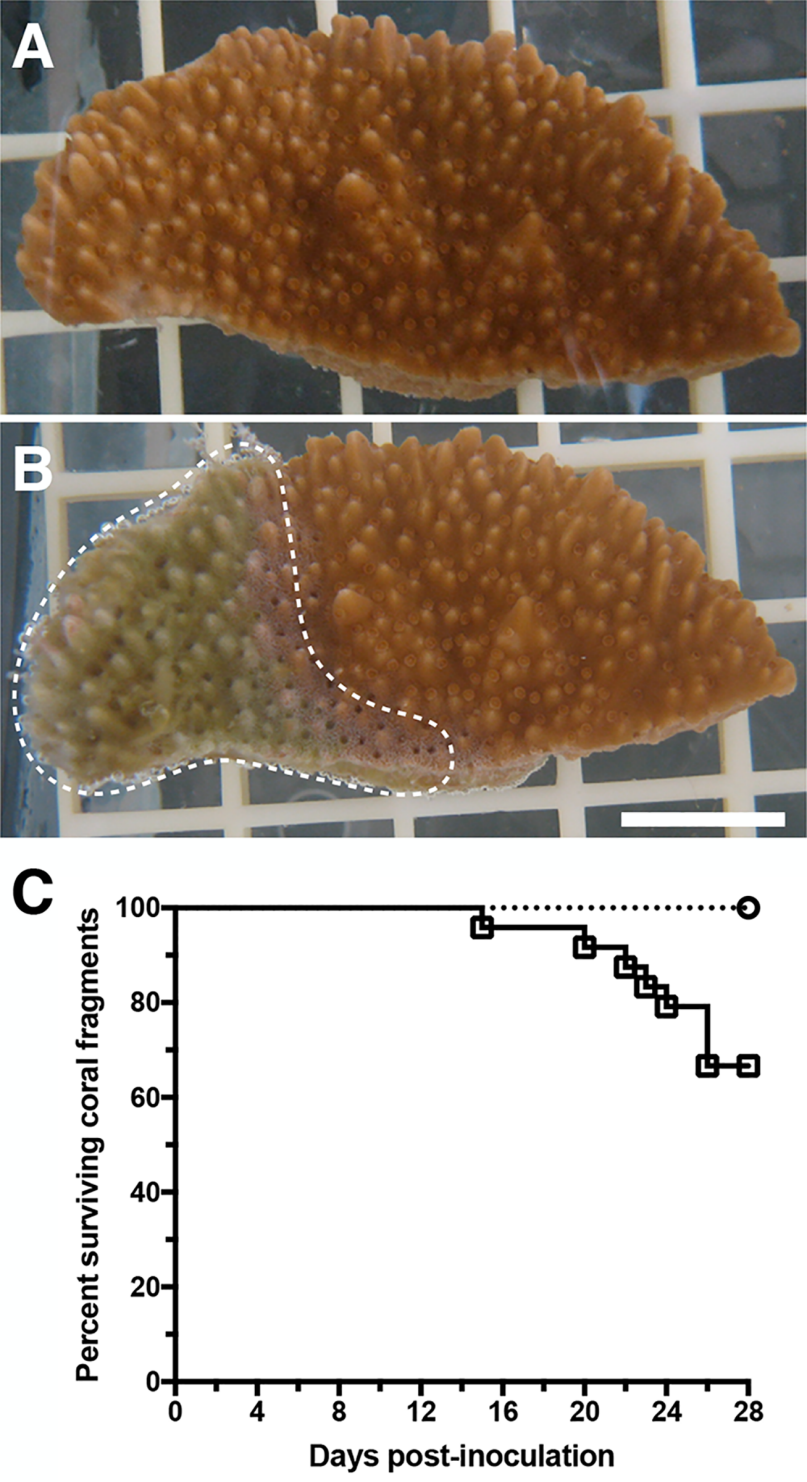
Example of aMWS in *Montipora capitata* caused by OCN003. (A) *M*. *capitata* before inoculation. (B) *M*. *capitata* 21 days post-inoculation with OCN003 displaying aMWS (white dashed line). The white scale bar represents one cm. (C) Kaplan–Meier survival curve of *M*. *capitata* fragments exposed to OCN003 (solid line with open black squares) or the control bacterium, OCN004 (dotted line with open circles), at 25°C (McNemar’s test, *n* = 24 each, *p* = 0.01). The concentration of bacteria used was 10^8^ CFU/ml of seawater.

The novel bacterial species *Pseudoalteromonas piratica* strain OCN003 [25]; hereafter OCN003) was originally isolated from an *M*. *capitata* colony displaying aMWS [24]. Due to the abundance of members from the *Pseudoalteromonas* genus in aMWS-affected *M*. *capitata* tissue, OCN003 was assessed for virulence against *M*. *capitata*. When healthy fragments of *M*. *capitata* were exposed to OCN003, 33% of the coral fragments developed acute tissue loss (aMWS) in an average of 22 days post-inoculation (McNemar’s test, *n* = 24, *p* = 0.01; Fig 1). The lesions induced by OCN003 in laboratory fragments were visually similar to aMWS observed on *M*. *capitata* both in the field and during previous laboratory experiments [7,14]. As controls, healthy *M*. *capitata* fragments were exposed to filtered seawater (FSW) or inoculated with the negative-control bacterium, *Alteromonas* sp. strain OCN004 [13], neither of which induced tissue loss (*n* = 24 per treatment). Using these two controls indicates that neither the microflora harbored by coral from the field nor the addition of a high concentration of control bacteria were sufficient to induce tissue loss lesions.

Prior studies defining coral pathogens as etiological agents of disease demonstrated the successful re-isolation of the pathogenic strain from experimentally infected coral fragments [13–15,19]. To facilitate re-isolation and ensure that the re-isolated bacteria were derived from the laboratory stock culture, OCN003 was genetically tagged with a non-self-transmissible plasmid, pRL1383a, as previously described [13]. The infection rates between the wild type OCN003 and tagged OCN003 were not significantly different (conducted simultaneously; Mantel-Cox test, *n* = 20, *p* = 0.98), with each bacterium inducing aMWS in 20% of the fragments after an average of 19 days post-inoculation. The tagged OCN003 was re-isolated from all experimentally infected fragments that developed aMWS (*n* = 20), using previously described methods [13]. Collectively, these results demonstrated that OCN003 was isolated from diseased coral, grown in pure culture, used to experimentally infect laboratory specimens, and was re-isolated from infected specimens. This fulfillment of Koch’s postulates of disease causation indicates that OCN003 is an etiological agent of aMWS.

### *P*. *piratica* strain OCN003 induces the switch from cMWS to aMWS in *M*. *capitata*

**Fig 2:**
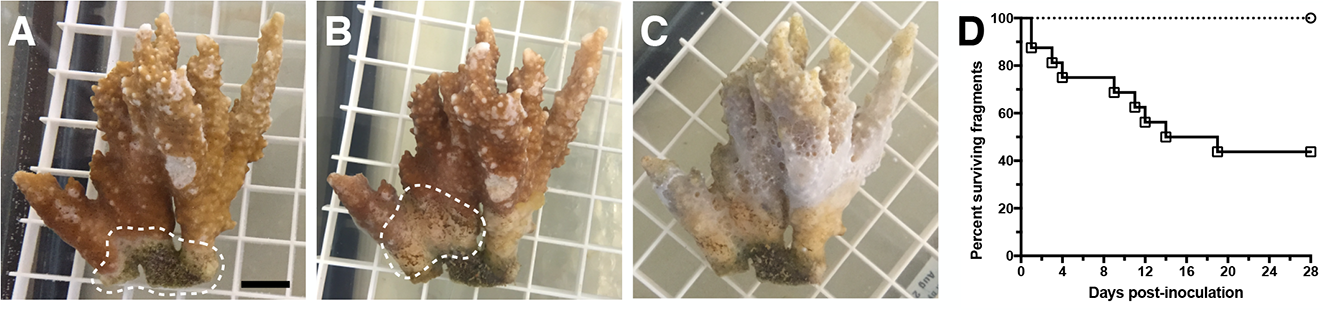
Example of OCN003 inducing the switch to aMWS on a *Montipora capitata* fragment with cMWS. (A) *M*. *capitata* with cMWS (white dashed line) before inoculation. (B) *M*. *capitata* three days after inoculation with OCN003, showing progressing tissue loss (aMWS; white dashed line). (C) *M*. *capitata* five days after inoculation with OCN003, displaying a complete loss of healthy tissue. The black scale bar represents one cm. (D) Kaplan–Meier survival curve of cMWS-affected *M*. *capitata* fragments exposed to OCN003 (solid line with open black squares) or the control bacterium, OCN004 (dotted line with open circles), at 25°C (McNemar’s test: *n* = 16 for each, *p* < 0.01). The concentration of bacteria used was 10^8^ CFU/ml of seawater.

In the field, *M*. *capitata* colonies with cMWS can suddenly display acute tissue loss that resembles aMWS [7]. This switch from a cMWS to aMWS could be due to many factors, one of which is the onset of a new or additional infection. To investigate whether OCN003 could induce a switch from a cMWS to aMWS, *M*. *capitata* fragments displaying cMWS were collected from the field and inoculated with OCN003. Following inoculation with OCN003, 56% of fragments with pre-existing cMWS switched to aMWS in an average of eight days post-inoculation (McNemar’s test: *n* = 16, *p* < 0.01; Fig 2). Following the OCN003-induced switch from cMWS to aMWS in the laboratory, the newly formed tissue loss lesions appeared visually similar to aMWS observed in the field and in the laboratory infections described above [7]; S2 Fig). The genetically-tagged OCN003 strain used above was also able to cause a switch from cMWS to aMWS at levels similar to the wild type (Mantel-Cox test: *n* = 6, *p* = 0.92), and could be re-isolated from all *M*. *capitata* fragments that switched from cMWS to aMWS. The FSW or OCN004 negative controls did not induce a switch from cMWS to aMWS (*n* = 16 per treatment; S3 Fig). The minimum dose of OCN003 required to induce the cMWS to aMWS switch was then determined by conducting infection trials on fragments with cMWS with inoculum concentrations ranging from 10^4^ to 10^8^ CFU/ml of tank water. After inoculation with 10^4^ and 10^5^ CFU of OCN003 per ml of tank water, 0% and 18% of fragments with cMWS developed aMWS, respectively. Therefore, the minimum dose required to induce the switch from cMWS to aMWS was found to be between 10^4^ and 10^5^ CFU/ml (*n* = 11). These results collectively fulfill Koch’s postulates to confirm that OCN003 acts as a pathogen to induce the switch from cMWS to aMWS.

### Motility is required for OCN003 to induce the cMWS to aMWS switch

**Fig 3:**
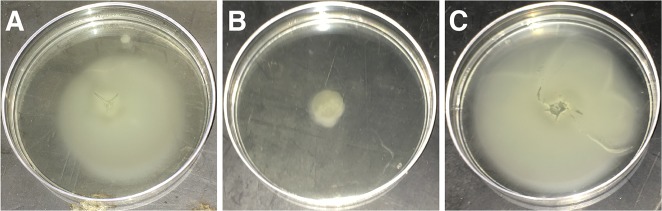
Semi-solid (0.15% agar) assay confirming motility of wild type OCN003 cells (A), the lack of motility of the OCN003 Δ*fliF*::*bla* mutant cells (B), and the restored motility of the complemented OCN003 Δ*fliF*::*bla* mutant cells (C).

**Fig 4:**
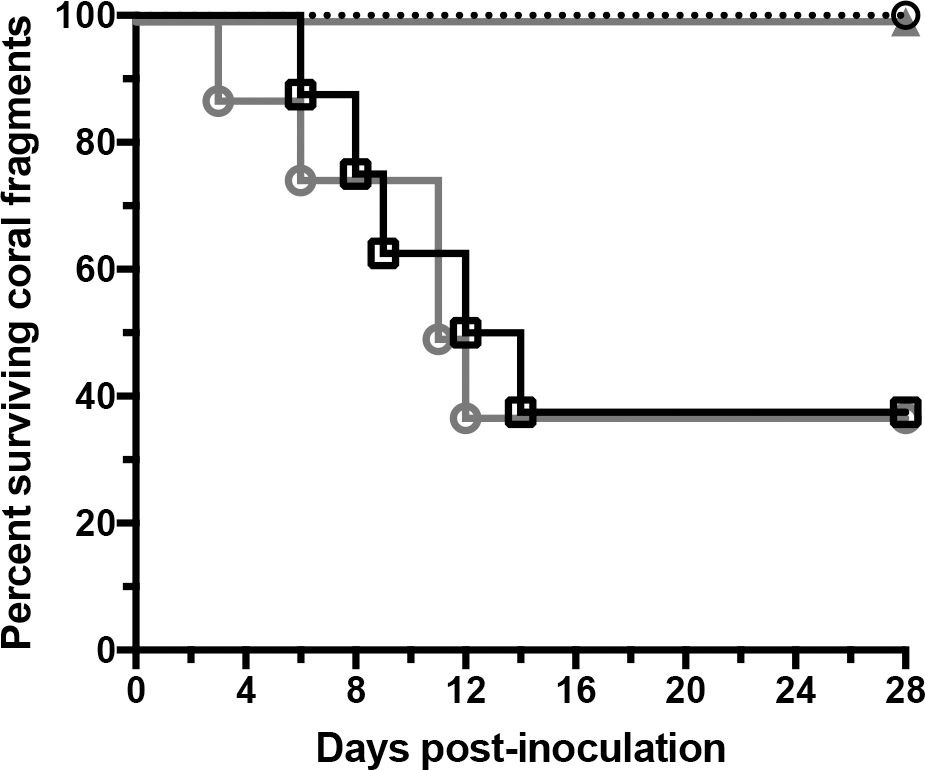
Kaplan–Meier survival curve of cMWS-affected *M*. *capitata* fragments exposed to OCN003 (solid line with open black squares), OCN003 Δ*fliF*::*bla* (solid grey line with open grey circles), the complemented OCN003 Δ*fliF*::*bla* mutant (dotted line with open black circles), or the control bacterium, OCN004 (solid grey line with closed grey triangles), at 25°C (*n* = 8 for each). The concentration of bacteria used was 10^8^ CFU/ml of seawater.

It is possible that the addition of an infectious strain, rather than the action of the strain itself, is able to induce the switch from cMWS to aMWS, thus, assessment of a mechanistic basis of virulence underlying the switch between cMWS and aMWS is pertinent. To assess a potential virulence factor of OCN003 contributing to its ability to induce the switch from cMWS to aMWS, a non-motile OCN003 mutant was created. Previous studies analyzing motility as a requirement for infection have demonstrated that a *flhA* mutant in *V*. *coralliilyticus* strain YB2 failed to form a flagellum and was incapable of infecting the coral *Pocillopora damicornis* [37]. In contrast, non-motile mutant strains of the human pathogen *Vibrio cholerae* were shown to remain infectious [38]. Rather than remove the flagellum as was previously done in *V*. *coralliilyticus* strain YB2 [37], a mutation abolishing flagellar movement was constructed in OCN003 to preserve the cell's morphology but not propel the organism. Previous work has shown that flagella can be quite immunogenic [39–41], so the introduction of two simultaneous phenotypic changes stemming from one mutation, the lack of a flagellum and abrogation of motility, would introduce additional difficulty into the data interpretation. Therefore, the *fliF* homolog in OCN003, which encodes a flagellar motor protein, was deleted to create a non-motile mutant that remained flagellated. Deletion of *fliF* in other bacteria results in the production of a non-functional flagellum, which abolishes bacterial motility and potentially impairs adhesion to the host [42,43]. The non-motile strain of OCN003 was created by replacing all but the first and last 18 nucleotides of the coding region of the *fliF* homolog in OCN003 [AIY66328] with the *bla* gene, which confers resistance to the antibiotic ampicillin. The resulting strain, OCN003 Δ*fliF*::*bla*, displayed wild type physiology, growth rate, and retained the ability to form a biofilm (S4 Fig). Though OCN003 Δ*fliF*::*bla* formed a flagellum (S5 Fig), it was non-functional and the mutant could no longer swim as confirmed by light microscopy and semi-solid agar assays (Fig 3). When the OCN003 Δ*fliF*::*bla* strain was used to infect cMWS-affected *M*. *capitata* fragments, the mutant was incapable of inducing the switch from cMWS to aMWS (Mantel-Cox test: *n* = 8, *p* < 0.01). The introduction of a plasmid carrying a functional copy of *fliF* into the OCN003 Δ*fliF*::*bla* mutant complemented the mutation and restored its ability swim and cause the switch from cMWS to aMWS at rates that were similar to wild type (Fig 4; Mantel-Cox test: *n* = 8, *p* = 0.90). The complemented OCN003 Δ*fliF*::*bla* mutant was also successfully re-isolated from all of the experimentally infected fragments. These results fulfill Koch’s molecular postulates for the requirement of *fliF*, and motility by extension, for proper virulence of OCN003 in the cMWS to aMWS switch. This indicates that wild type OCN003, not just the presence of viable OCN003 cells, is required to induce the switch from cMWS to aMWS.

### *Pseudoalteromonas piratica* strains were isolated from diseased *M*. *capitata* fragments collected during the 2010 aMWS outbreak

**Fig 5:**
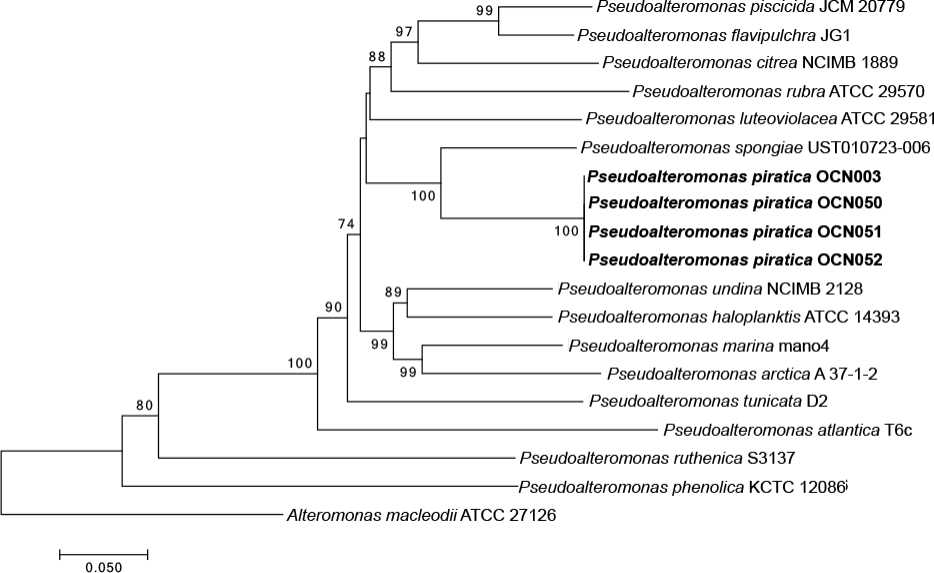
Neighbor-joining dendrogram showing the estimated phylogenetic relationships between the *Pseudoalteromonas piratica* strains OCN003 (type strain), OCN050, OCN051, OCN052 (strains isolated during the 2010 aMWS outbreak), and related *Pseudoalteromonas* spp. based upon a multi-locus sequence analysis. Analysis was based on the sequences of the housekeeping genes *recA*, *gapA*, and *ftsZ*. *Alteromonas macleodii* ATCC 27126 was chosen as the outgroup. The scale bar represents five nucleotide substitutions per 100 nucleotides. Bootstrap values >70% (500 replicates) are indicated at nodes.

Based on the finding that OCN003 can cause aMWS, it is possible that coral colonies displaying aMWS in the field during outbreaks were infected by OCN003. To determine whether OCN003, or similar *P*. *piratica* strains, may have been involved in the March 2010 aMWS outbreak that affected several hundred colonies of *M*. *capitata* [7], bacteria were cultured from the mucus of infected colonies and identified. A total of 265 bacterial isolates were cultured from four aMWS-infected coral fragments collected during the outbreak. These isolates were screened by PCR using previously described primers (OCN008-42310-F and OCN008-43080-R) specific to *V*. *coralliilyticus* strain OCN008, a pathogen known to cause aMWS in *M*. *capitata* in Kāne‘ohe Bay [14], or primers that selectively amplify a unique region of the OCN003 genome (003unique-F and 003unique-R). The unique region used for OCN003 identification was found by searching the genome [28] for a large intragenic region between convergent genes that was not similar to any sequences present in the NCBI database (see Materials and Methods for a description). Five isolates from the 2010 outbreak grew on *Vibrio-*selective TCBS agar, but none yielded a PCR product using the OCN008-specific primers. In contrast, three of the 265 isolates yielded a PCR product when screened with the OCN003-specific primers. The 16S rRNA gene sequences from each of the three OCN003-like isolates, referred to as OCN050, OCN051, and OCN052, were identical to the sequence from OCN003 and all three isolates clustered with OCN003 during MLSA comparison, which supports their classification as *P*. *piratica* strains OCN050, OCN051, and OCN052 (Fig 5). Based on the similarity of these isolates, the remainder of the study utilized OCN050 as a representative isolate from the outbreak.

**Fig 6:**
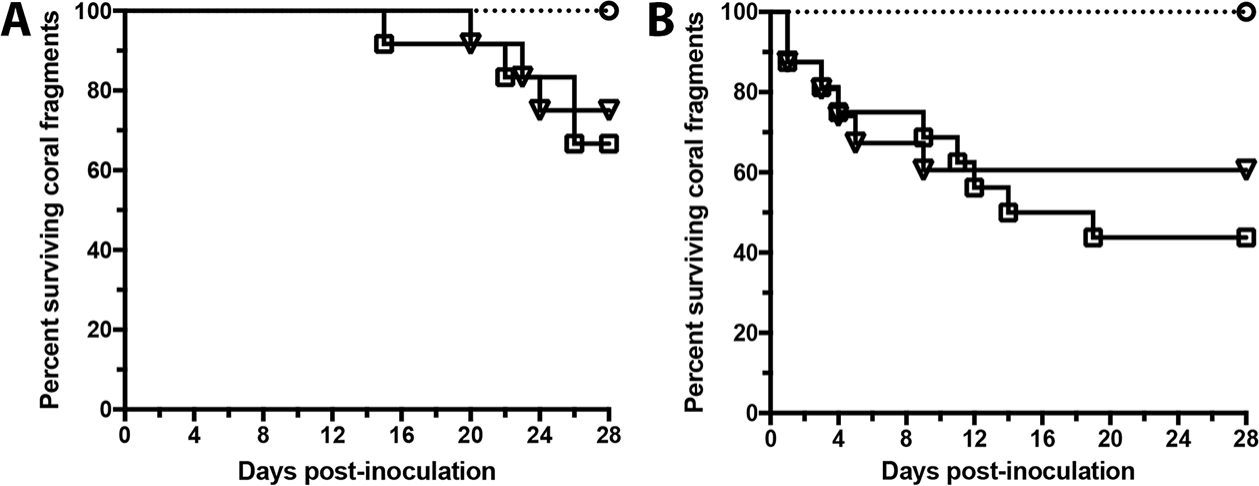
Kaplan–Meier survival curves of healthy (A) and cMWS-affected (B) *Montipora capitata* fragments exposed to OCN003 (solid line with open black squares), OCN050 (solid line with open black triangles; Mantel-Cox test: *n =* 12, *p* = 0.71 (A); Mantel-Cox test: *n* = 16, *p* = 0.50 (B)), or the control bacterium, OCN004 (dotted line with open circles), at 25°C (*n* = 12 (A) or *n* = 16 (B) for each). The concentration of bacteria used was 10^8^ CFU/ml of seawater.

While sequence identity indicated that strains OCN003 and OCN050 are genetically similar, the next step was to determine whether OCN050 could also induce aMWS in healthy *M*. *capitata* fragments and induce the cMWS to aMWS switch in a manner similar to OCN003. During infection trials conducted like those above, OCN050 infected healthy fragments of *M*. *capitata* at levels that were similar to those of OCN003, 25% and 33%, respectively (Mantel-Cox test: *n =* 12, *p* = 0.71; Fig 6). None of the negative control fragments displayed any signs of tissue loss following exposure to FSW or the negative control bacterium, OCN004 (*n* = 12 per treatment). In addition, OCN050 induced a switch from cMWS to aMWS in 38% of the exposed cMWS-affected fragments (McNemar’s test: *n* = 16, *p* = 0.04), which was not significantly different from the OCN003-mediated switch from cMWS to aMWS (Mantel-Cox test: *n* = 16, *p* = 0.50; Fig 6). These data indicate that OCN003 and OCN050 infect *M*. *capitata* fragments similarly and can induce the switch from cMWS to aMWS in laboratory conditions. Taken together, OCN003 and OCN003-like strains are etiological agents of acute tissue loss disease in *M*. *capitata* and may have been involved in the 2010 aMWS outbreak.

### *P*. *piratica* strains and *V*. *coralliilyticus* strain OCN008 induce the cMWS to aMWS switch at different rates

**Fig 7:**
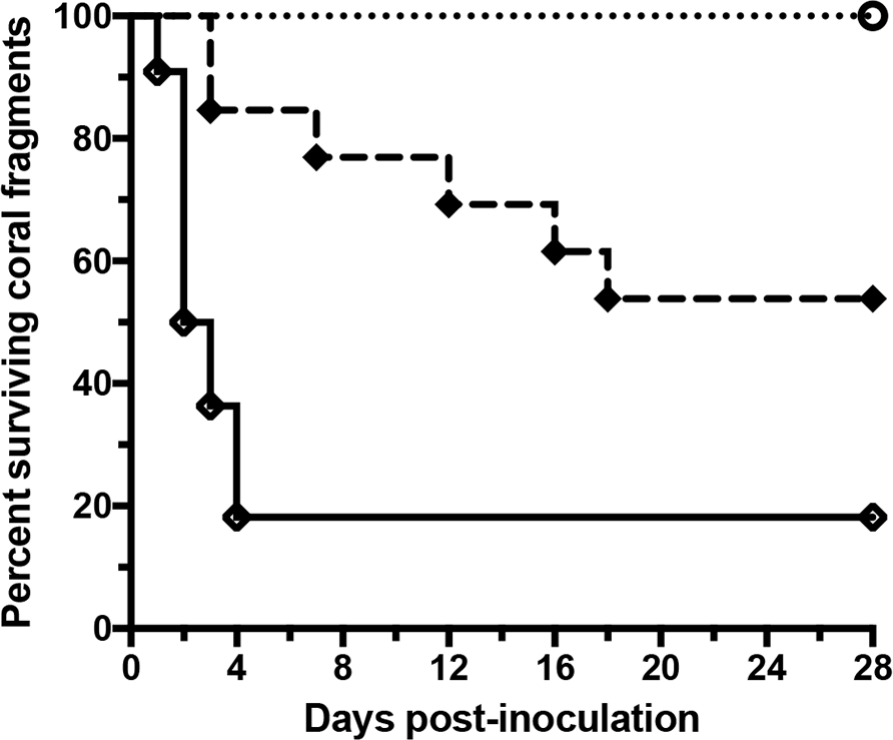
Kaplan–Meier survival curve of healthy or cMWS-affected *M*. *capitata* fragments exposed to OCN008 or the control bacterium, OCN004, at 25°C. The solid line with open black rhombi represents healthy coral fragments exposed to OCN008 (*n* = 22); the dashed line with closed black rhombi represents cMWS-affected coral fragments exposed to OCN008 (*n* = 13); the dotted line with open circles represents healthy or cMWS-affected coral fragments exposed to OCN004 (*n* = 22 or *n* = 13, respectively). The concentration of bacteria used was 10^8^ CFU/ml of seawater.

Like OCN003, *Vibrio coralliilyticus* strain OCN008 has fulfilled Koch’s postulates as an etiological agent of aMWS for *M*. *capitata* [14]. It is possible that any bacterial agent that can cause aMWS can induce a switch from cMWS to aMWS, and that this is not an OCN003-specific interaction with *M*. *capitata*. To assess the specificity of two aMWS-causing pathogens to induce the switch from cMWS to aMWS, cMWS-affected *M*. *capitata* fragments were exposed to OCN008. When healthy coral fragments were exposed to OCN008 as a primary pathogen, 80% of the fragments developed aMWS in an average of two days post-inoculation (McNemar’s test: *n =* 22, *p* < 0.01). However, when aliquots of the same OCN008 cultures were used to inoculate *M*. *capitata* with cMWS, only 46% of the fragments developed aMWS in an average of 10 days post-inoculation (McNemar’s test: *n =* 13, *p* = 0.04; Fig 7). In contrast, cMWS-affected fragments from the same diseased colonies were more susceptible to OCN003, which was able to induce the switch to aMWS in 62% of specimens in an average six days post-inoculation during concurrent infection trials. Analysis of the infection rates of OCN003 on healthy and cMWS-affected *M*. *capitata* shows that its ability to infect fragments with pre-existing cMWS is significantly higher than its infection of healthy fragments (Mantel-Cox test: *n*_*1*_ = 16, *n*_*2*_ = 12, *p* = 0.03). The opposite result was found for OCN008, in which its ability to infect cMWS-affected *M*. *capitata* fragments was statistically lower than its infection of healthy fragments (Mantel-Cox test: *n*_*1*_ = 13, *n*_*2*_ = 22, *p* < 0.01). When healthy and cMWS-affected fragments were exposed to the control bacterium, OCN004, no fragments switched from cMWS to aMWS. Taken together, these results demonstrate that OCN008 infects healthy *M*. *capitata* more effectively while OCN003 is more effective at infecting cMWS-affected colonies.

## Discussion

Here we describe a novel *Pseudoalteromonas* species, *P*. *piratica* strain OCN003, which acts as another etiological agent of aMWS on healthy *M*. *capitata* coral and as a pathogen that can induce the switch from cMWS to aMWS. Additional strains of *P*. *piratica*, were isolated from *M*. *capitata* displaying acute tissue loss collected during a 2010 aMWS outbreak. These *P*. *piratica* strains also infected healthy *M*. *capitata* fragments in controlled laboratory experiments, suggesting that *P*. *piratica* may have been involved in the 2010 aMWS outbreak in Kāne‘ohe Bay. Additionally, motility was required as a virulence mechanism influencing the ability of OCN003 to induce the switch from cMWS to aMWS. This work also demonstrates that two different bacteria (*P*. *piratica* strain OCN003 and *V*. *coralliilyticus* strain OCN008) can elicit the same disease signs (aMWS) in a single coral species (*M*. *capitata*). Additional undiscovered pathogens may be capable of causing aMWS and other coral diseases with known etiologies.

The two pathogens, OCN003 and OCN008, displayed very different levels of virulence on healthy vs. cMWS-affected *M*. *capitata*. OCN003 infected 33% of healthy fragments roughly 22 days post-inoculation, but infected 56% of cMWS-affected fragments about eight days post-inoculation. In contrast, OCN008 infected 80% of healthy fragments two days post-infection but only infected 46% of cMWS-affected fragments about ten days post-infection. The differences in rates of infection between these bacteria on healthy vs. cMWS-affected *M*. *capitata* suggests that some component of either the coral or the pathogen are modulating virulence under these conditions. The microflora present in healthy coral mucus confer some level of protection against bacterial infection either by competing for space or nutrients or by producing antimicrobial compounds to inhibit pathogen growth [44–49]. Gochfeld and Aeby (2008) [50] demonstrated that crude aqueous extracts from healthy *M*. *capitata* colonies from Hawaiian reefs including Kāne‘ohe Bay, exhibited a significant amount of antibacterial activity with a high degree of selectivity; certain bacteria were inhibited whereas others were not. Bacteria, including OCN003 and OCN008, vary in their sensitivity to antimicrobial compounds. OCN003 is resistant to kanamycin (100 μg) whereas OCN008 is resistant to both kanamycin (25 μg) and ampicillin (800 μg) [14,25]. Perhaps OCN008 and OCN003 differ in their sensitivities to antibiotics produced by the microflora on *M*. *capitata*, which could affect their respective capacities to infect.

OCN008 and OCN003 differed significantly in their ability to infect a compromised host (coral with cMWS). Studies have shown that the bacterial communities in coral mucus change significantly during infection [51–56]. This pattern holds true for *M*. *capitata*; colonies with cMWS have different bacterial communities compared to uninfected colonies [24]. The shift in the coral-associated microflora could differentially modulate the infectivity of these pathogens, increasing the ability of OCN003 to infect and decreasing the infectivity of OCN008. OCN003 may be able to infect cMWS-affected *M*. *capitata* colonies more readily because some members of the bacterial microflora present in healthy mucus inhibited OCN003 infection, but not OCN008 infection, and were absent from diseased fragments. For example, Smith (2008) found that *Streptomyces*, a genus known for their capacity to produce antibiotics [57], was a dominant component of the culturable bacterial community in healthy *M*. *capitata* but were absent in cMWS-affected coral. Why OCN008 and OCN003 differ in their abilities to infect healthy and cMWS-affect *M*. *capitata* is currently unknown but this study raises some interesting hypotheses that warrant further investigation.

## Conclusions

This work describes the first non-*Vibrio* etiological agent of acute tissue loss disease in *M*. *capitata*, *Pseudoalteromonas piratica* strain OCN003, and is also the first member of this genus confirmed as a coral pathogen. We show that OCN003 can infect healthy *M*. *capitata* as an etiological agent of aMWS but was able to infect corals at a significantly higher rate when inoculated onto *M*. *capitata* displaying chronic MWS (cMWS). We contrast this with the known pathogen *V*. *coralliilyticus* strain OCN008, which infects compromised coral (cMWS) at a significantly lower rate than healthy coral. The different levels of virulence displayed by these two bacterial pathogens toward *M*. *capitata* when used to induce aMWS in either healthy or cMWS-affected fragments, suggests that some component of either the coral or the pathogen can modulate virulence in this system. This work provides the first demonstration of two different genera of bacterial pathogens that act as etiological agents producing identical lesions in corals.

## Supporting information

S1 Fig Schematic of the coral infection protocol utilized in this work. The organisms used for infection are grown to the optical density specified, washed with artificial seawater (ASW), and inoculated into temperature-controlled aquaria housing healthy (right track) fragments of *M*. *capitata* or fragments displaying chronic *Montipora* white syndrome (cMWS; denoted by a dark spot on the fragment). Following inoculation, fragments were monitored for the onset of tissue loss similar to acute *Montipora* white syndrome (aMWS), which can result in exposure of the white coral skeletons. Prior to complete lysis, the remaining tissue from aMWS infectioned fragments was harvested, homogenized, and either plated on appropriate media to recover the pathogen and used to identify desired bacteria.
(TIF)

S2 Fig Time course of the progression of aMWS following the OCN003-induced switch to aMWS of *Montipora capitata* fragment with cMWS. (A) *M*. *capitata* with cMWS lesion (white dashed line) before inoculation. *M*. *capitata* fragment two days (B), three days (C), four days (D), five days (E), and six days (F) post-inoculation with OCN003 displaying a progressing aMWS (white arrows). The black scale bar represents one cm. The concentration of bacteria used was 10^8^ CFU/ml of seawater.
(TIFF)

S3 Fig Example of a *Montipora capitata* fragment exhibiting cMWS lesions following inoculation with the control bacterium, OCN004. (A) *M*. *capitata* with cMWS before inoculation (white line). (B) *M*. *capitata* 28 days post-inoculation with OCN004, showing persistent cMWS (white line). The black scale bar represents one cm. The concentration of bacteria used was 10^8^ CFU/ml of seawater.
(TIFF)

S4 Fig Biofilm formation of OCN003 (dark grey bars), the OCN003 Δ*fliF*::*bla* mutant (grey bars), and the complemented OCN003 Δ*fliF*::*bla* mutant (white bars). The graph shows the average absorbance for each strain with error bars representing the standard error of the mean (SEM). The assay was performed at 24 h, 48 h, 72 h, and 96 h post-inoculation.
(TIFF)

S5 Fig Electron micrographs of contrasted preparations of OCN003 (A), the OCN003 Δ*fliF*::*bla* mutant (B), and the complemented OCN003 Δ*fliF*::*bla* mutant (C) showing the presence of a polar flagellum (arrow). Cells used for analysis were deposited on Formvar-coated copper grids and contrasted with 1% uranyl acetate for viewing on a Hitachi HT7700 TEM at 100 kV. Images were captured with an AMT XR-41B 2k x 2k CCD camera. Scale bar represents one μm.
(TIFF)
