# Supplementary figures and images for: Pseudoalteromonas piratica strain OCN003 is a coral pathogen that causes a switch from chronic to acute Montipora white syndrome in Montipora capitata
Source: PLoS One. 2017 Nov 16;12(11):e0188319. doi: 10.1371/journal.pone.0188319 (PMC5690655; doi:10.1371/journal.pone.0188319)

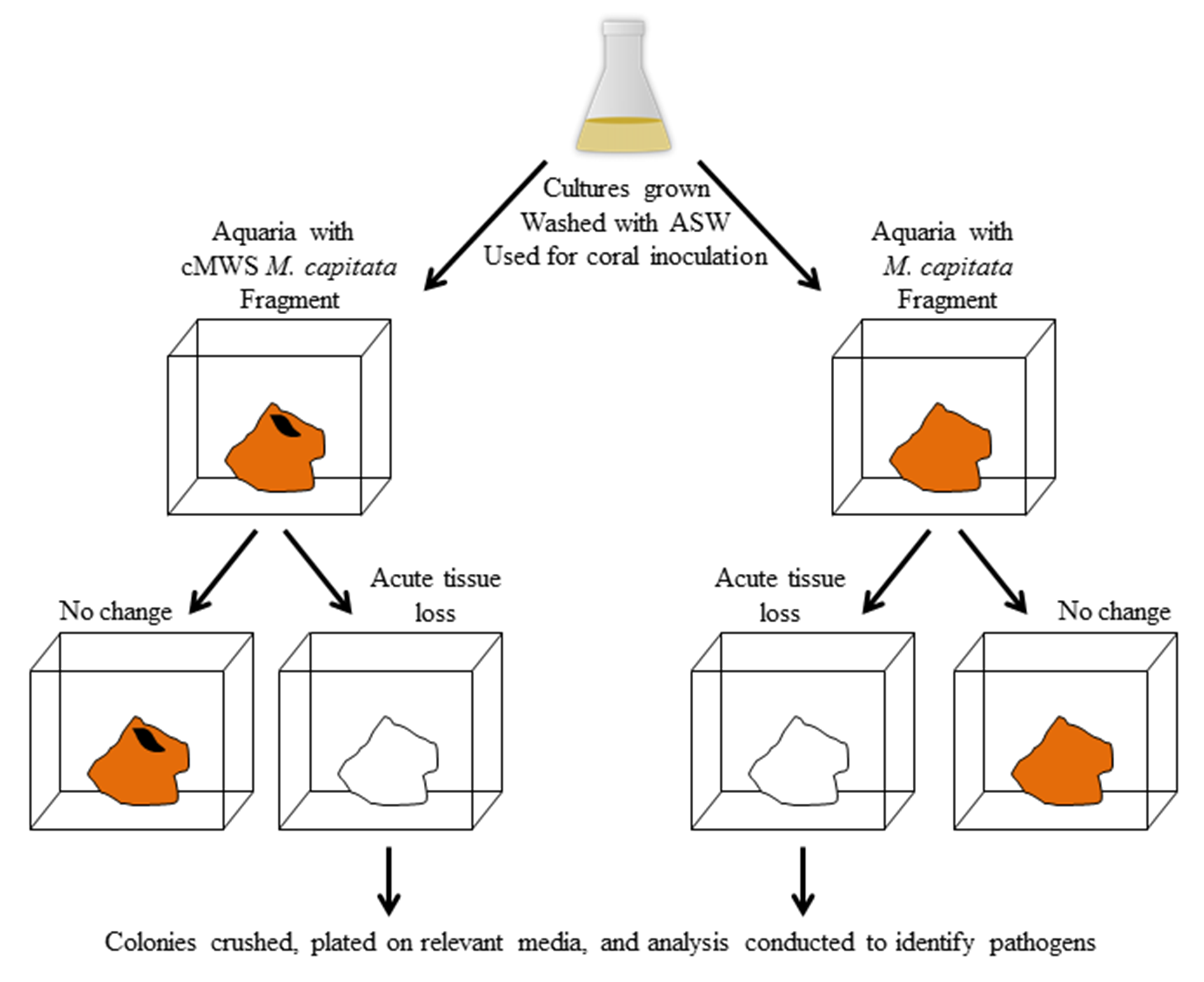

Supplement: S1 Fig — The organisms used for infection are grown to the optical density specified, washed with artificial seawater (ASW), and inoculated into temperature-controlled aquaria housing healthy (right track) fragments of M. capitata or fragments displaying chronic Montipora white syndrome (cMWS; denoted by a dark spot on the fragment). Following inoculation, fragments were monitored for the onset of tissue loss similar to acute Montipora white syndrome (aMWS), which can result in exposure of the white coral skeletons. Prior to complete lysis, the remaining tissue from aMWS infectioned fragments was harvested, homogenized, and either plated on appropriate media to recover the pathogen and used to identify desired bacteria. (TIF) [file pone.0188319.s001.tif]

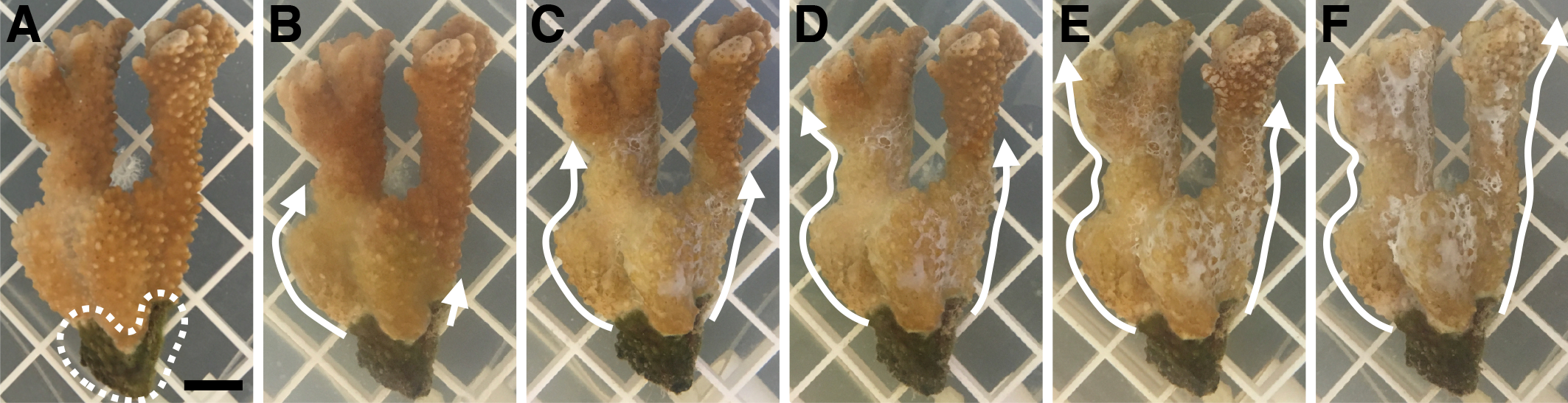

Supplement: S2 Fig — (A) M. capitata with cMWS lesion (white dashed line) before inoculation. M. capitata fragment two days (B), three days (C), four days (D), five days (E), and six days (F) post-inoculation with OCN003 displaying a progressing aMWS (white arrows). The black scale bar represents one cm. The concentration of bacteria used was 108 CFU/ml of seawater. (TIFF) [file pone.0188319.s002.tiff]

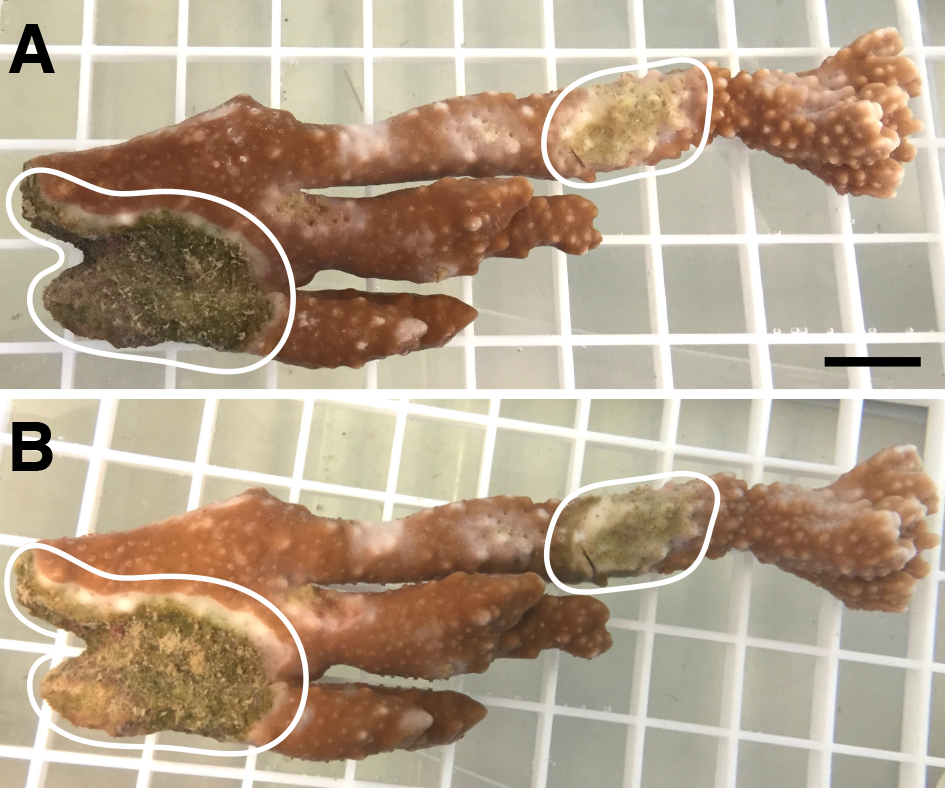

Supplement: S3 Fig — (A) M. capitata with cMWS before inoculation (white line). (B) M. capitata 28 days post-inoculation with OCN004, showing persistent cMWS (white line). The black scale bar represents one cm. The concentration of bacteria used was 108 CFU/ml of seawater. (TIFF) [file pone.0188319.s003.tiff]

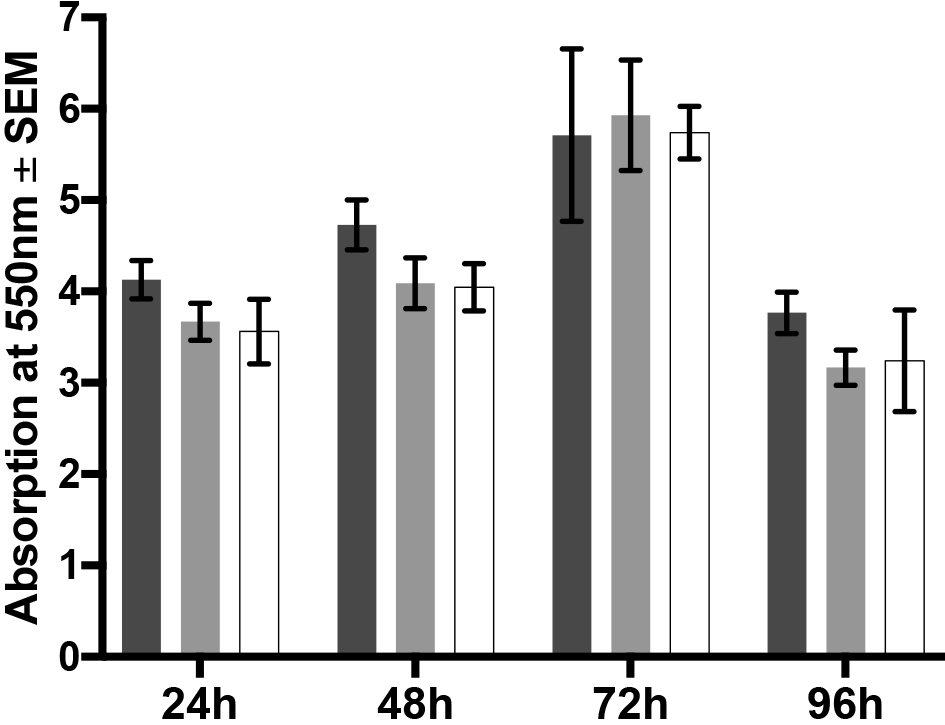

Supplement: S4 Fig — The graph shows the average absorbance for each strain with error bars representing the standard error of the mean (SEM). The assay was performed at 24 h, 48 h, 72 h, and 96 h post-inoculation. (TIFF) [file pone.0188319.s004.tiff]

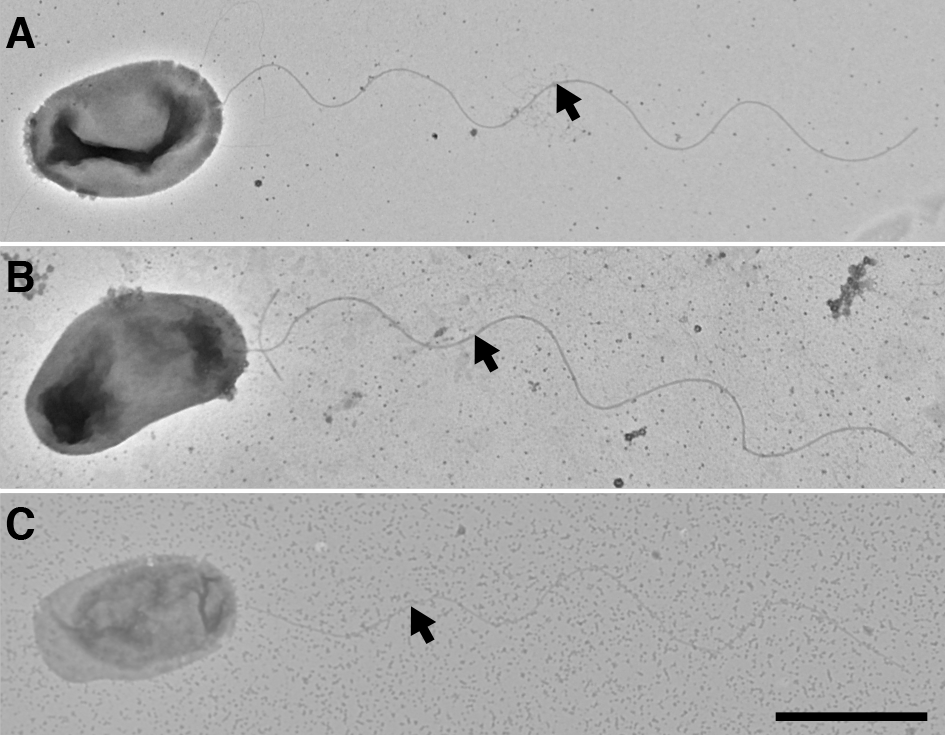

Supplement: S5 Fig — Electron micrographs of contrasted preparations of OCN003 (A), the OCN003 ΔfliF::bla mutant (B), and the complemented OCN003 ΔfliF::bla mutant (C) showing the presence of a polar flagellum (arrow). Cells used for analysis were deposited on Formvar-coated copper grids and contrasted with 1% uranyl acetate for viewing on a Hitachi HT7700 TEM at 100 kV. Images were captured with an AMT XR-41B 2k x 2k CCD camera. Scale bar represents one μm. (TIFF) [file pone.0188319.s005.tiff]
